# Modeling truncated and censored data with the diffusion model in Stan

**DOI:** 10.3758/s13428-025-02822-z

**Published:** 2026-01-20

**Authors:** Franziska Henrich, Karl Christoph Klauer

**Affiliations:** Department of Psychology, University of Freiburg, Engelbergerstraße 41, 79106 Freiburg, Germany

**Keywords:** Ratcliff diffusion model, Stan, Truncation, Censoring

## Abstract

Reaction time data in psychology are frequently censored or truncated. For example, two-alternative forced-choice tasks that are implemented with a response window or response deadline give rise to censored or truncated data. This must be accounted for in the data analysis, as important characteristics of the data, such as the mean, standard deviation, skewness, and correlations, can be strongly affected by censoring or truncation. In this paper, we use the probabilistic programming language Stan to analyze such data with Bayesian diffusion models. For this purpose, we added the functionality to model truncated and censored data with the diffusion model by adding the cumulative distribution function for reaction times generated from the diffusion model and its complement to the source code of Stan. We describe the usage of the truncated and censored models in Stan, test their performance in recovery and simulation-based calibration, and reanalyze existing datasets with the new method. The results of the recovery studies are satisfactory in terms of correlations ($r=.93 - 1.00$), coverage (93–95% of true values lie in the 95% highest density interval), and bias. Simulation-based calibration studies suggest that the new functionality is implemented without errors. The reanalysis of existing datasets further validates the new method.

## Introduction

Truncation and censoring frequently occur in psychological data collection (Barchard & Russell, 2024; Ulrich & Miller, 1994). For reaction time data, truncated and censored data regularly arise in psychological studies as a consequence of using response windows or deadlines. These are sometimes introduced in the analysis of data to exclude reaction times that appear too short or too long, but they are also sometimes already built into the study procedures to push participants to respond within a specific temporal window. For example, in the area of social cognition (Carlston et al., 2024), experiments frequently use two-alternative forced-choice tasks to measure implicit mechanisms in stereotyping and prejudice. To reveal fast-acting, possibly implicit processes in stereotyping and prejudice, researchers focus on the outcomes of fast automatic processing at the expense of slow controlled processes. One way to facilitate this is to introduce a response window, forcing participants to respond quickly.

Below, we reanalyze such data stemming from the First-Person Shooter Task (FPST, Correll et al., 2002). In this and the similar Weapon Identification Task (WIT, Payne, 2001), participants are to discriminate between a weapon and a harmless object, independent of the skin color of a person shown before (WIT) or in parallel (FPST) with the target object. One central finding is that a harmless object is more often mistaken for a weapon when the person is Black than when the person is White. Moreover, participants are faster to correctly detect a weapon when the person is Black than when the person is White (e.g., Payne, 2001; 2006). Thus, there is *racial bias* in the accuracy data as well as in the reaction time data.

**Fig. 1:**
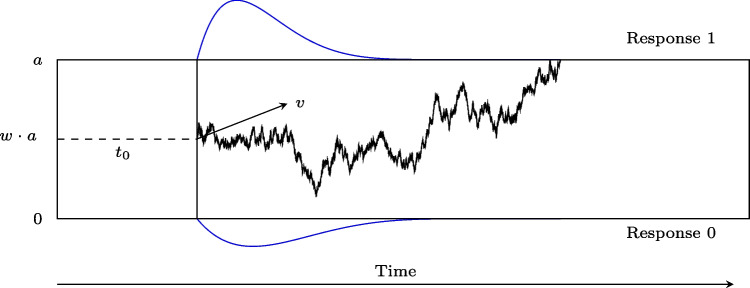
Realization of a four-parameter diffusion process modeling the binary decision process. *Note.* The parameters are the *boundary separation* *a* for two response alternatives, the *relative starting point* *w*, the *drift rate* *v*, and the *non-decision time* $t_0$. The decision process is illustrated as a *jagged line* between the two boundaries. The predicted distributions of the reaction times are depicted in *blue*

To make sure that participants respond as fast as possible, response deadlines are often implemented in the task. If participants do not respond prior to a certain response deadline, the trial is terminated and excluded from subsequent analyses (e.g., Lambert et al., 2003; Payne, 2001). Typical response deadlines in such tasks range from 500 ms (e.g., Todd et al., 2016) to 850 ms (e.g., Johnson et al., 2017).

Another use of response windows relies on the fact that the effects that are of interest are often considerably more pronounced in the accuracy data when a response window is in place. This has been found to increase the size and reliability of effects in some paradigms (Draine & Greenwald, 1998; Krause et al., 2012). Finally, as already mentioned, response windows are regularly imposed post-hoc in outlier analyses to exclude responses with implausibly short or long reaction times.

Depending on the implementation of the response window, two different types of data arise: truncated data or censored data. Since the effects of truncation or censoring on summary statistics such as mean, median, standard deviation, and skewness is regularly too large to ignore (Ulrich & Miller, 1994), data analysts are well advised to account for these effects. Here, we focus on analyses using diffusion models, which are frequently applied to data from two-alternative forced-choice tasks. For example, in the context of the FPST and the WIT, diffusion modeling has been employed by Correll et al. (2002); Payne (2001); Pleskac et al. (2018); Rivers (2017); Thiem et al. (2019); Todd et al. (2020).

Diffusion models model response times and responses simultaneously, thereby maximizing the use of available data. The basic diffusion model incorporates four parameters (Ratcliff, 1978), which can be interpreted in terms of psychological processes; an extended version of the diffusion model uses seven model parameters (Ratcliff & Rouder, 1998). A number of software packages allow one to estimate the parameters of the diffusion model such as dedicated modules implemented in Stan (Carpenter et al., 2017), JAGS (Wabersich & Vandekerckhove, 2013), WinBUGS (Vandekerckhove & Tuerlinckx, 2007), stand-alone software such as fast-dm (Voss & Voss, 2007), HDDM (Wiecki et al., 2013), HSSM (Fengler et al., 2013), or R-packages such as EMC2 (Stevenson et al., 2024), DMC (Heathcote et al., 2019), and ggdmc (Lin & Strickland, 2020), among others. However, only a few of these data-analytic solutions are able to directly model truncated or censored response time data. For example, the probabilistic programming language Stan does not have a built-in method to handle censoring or truncation with the diffusion model. To fill this gap, we added the functionality to deal with truncated and censored data in diffusion model analyses to Stan. This requires implementing the cumulative distribution function (CDF) of response-time distributions that arise under the diffusion model and its complement (CCDF) in Stan.

We chose Stan because of its usefulness and popularity as a free and open-source software package that provides users with many functions for Bayesian statistical inference and hierarchical modeling for a huge range of model families. Besides the diffusion model, numerous other models can be estimated in Stan, as many probability density functions like the ones for Bernoulli, beta, binomial, exponential, normal, and Poisson distributions, to name just a few, are implemented in Stan. These functions enable the user to choose the priors for the model parameters in a flexible manner. Furthermore, the probability density function (PDF) of the seven-parameter diffusion model is already available in this programming language (Henrich et al., 2023).

The goal of the present paper is to document and validate the new functionality to model truncated or censored data with the diffusion model in Stan using the CDF and CCDF functions. In the following sections, we provide a brief introduction to the diffusion model. Next, we elaborate on the notions of truncated and censored data and how they can be modeled in Stan. Following this, we conduct two validity checks for the new functionality: a simulation study showing good recovery for truncated and censored data, and a simulation-based calibration study. Finally, we present a reanalysis of existing datasets from the First-Person Shooter Task with a basic, a censored, and a truncated diffusion model.

## The diffusion model

The diffusion model (Ratcliff, 1978) is a member of the family of information accumulation models. It is widely used to model two-alternative forced-choice tasks by simultaneously modeling response time and responses (for a review, see Ratcliff et al., 2016).

In the basic model, four parameters describe the decision process (see Fig. 1): The process starts at a *relative starting point*, *w*, between the two response boundaries. Bits of information are noisily accumulated until one of the boundaries is reached, in which case the response associated with that boundary is initiated. The distance between both response boundaries is the *boundary separation*, *a*. The direction of the accumulation process is described by the *drift rate*, *v*, which corresponds to the average rate of information uptake. And finally, all processes that do not belong to the decision process itself, for example, the time taken for early perceptual encoding or production of the motor response, are summed in the *non-decision time*, $t_0$. The model predicts the reaction time distributions for the response associated with each boundary and the probabilities with which either response is made. Ratcliff and Rouder (1998) later extended the four-parameter diffusion model by adding three inter-trial variabilities (in *relative starting point*, *drift rate*, and *non-decision time*) to account for several reaction time patterns that could not be handled by the basic diffusion model. For example, when the relative starting point is set to 0.5, as is a priori plausible in many discrimination tasks when responses are coded as false versus correct, the basic diffusion model predicts the same response-time distributions for false and correct responses. In contrast, as explained by Ratcliff and Rouder (1998), the seven-parameter diffusion model allows one to account for error responses that are systematically faster (or slower) than correct responses.

Existing implementations of the diffusion model enable the estimation of four or seven parameters in both non-hierarchical and hierarchical settings, as well as in non-Bayesian and Bayesian contexts. However, only a few of the existing implementations of the diffusion model are able to directly model censored or truncated data arising from the use of response windows or response deadlines.^1^ Instead, many researchers use models that treat data as if they were not truncated or censored (e.g., Correll et al., 2015; Todd et al., 2020). In the following, we will elaborate on the notions of truncated and censored data and on how such data can be modeled with the diffusion model in Stan.

## Truncated and censored data

### Truncated data

Data are called *truncated* when there is no information available for analysis from trials with values larger (or smaller) than a right (or left) boundary. In our example of reaction time experiments, reaction time data are truncated if trials with reaction times outside the response window are excluded from the analysis. Not even a count of those omitted trials is kept.

Mathematically, truncation can be defined as follows: First, the notion of *cumulative distribution functions* is needed. A cumulative distribution function (CDF) of a real-valued random variable *X* evaluated at *x* is defined as the probability *P* that *X* takes a value less than or equal to *x*: $\text {CDF}(x) = P(X\le x)$.

Let *X* be a random variable, $\text {PDF}(x)$ its probability density function, and $\text {CDF}(x)$ its cumulative distribution function. Then, the PDF of *X* after truncating to the interval (*L*, *U*], such that $L<X\le U$, is defined as follows:

1 $$\begin{aligned} \text {PDF}(x \mid L<X\le U) = \frac{\text {PDF}(x)\cdot \mathbb {I}_{\{L<x\le U\}}}{\text {CDF}(U)-\text {CDF}(L)}, \end{aligned}$$

where $\mathbb {I}$ is the indicator function taking the value 1 if the condition in the parentheses holds and the value 0 otherwise.

In the case that *X* is truncated at only one side, its PDF is defined for left truncation as

2 $$\begin{aligned} \text {PDF}(x \mid L<X) = \frac{\text {PDF}(x)\cdot \mathbb {I}_{\{L<x\}}}{1-\text {CDF}(L)}, \end{aligned}$$

and for right truncation as

3 $$\begin{aligned} \text {PDF}(x \mid X\le U) = \frac{\text {PDF}(x)\cdot \mathbb {I}_{\{x\le U\}}}{\text {CDF}(U)} \end{aligned}$$

### Censored data

Data are *censored* when observations that are above or below a right or left boundary value are reported as occurrences of the event $(x>U)$, for *U* the right bound, or as occurrences of the event $(x\le L)$, for *L* the left bound, respectively. Like for truncated data, the range of the possible values is restricted, but the number of observations that fall outside the boundaries is kept, whereas in truncation, no count would be kept.

Let *X* be a random variable, $\text {PDF}_X(x)$, and $\text {CDF}_X(x)$ be the probability density function and the cumulative distribution function of *X*. Let *Z* be a second random variable that is censored in the interval (*L*, *U*]. Let *Z* take the value of *X* if a realization of *X* is within the boundaries, and the value $l\le L$ if it is smaller than the lower bound, and the value $u>U$ if it is larger than the upper bound:

4 $$\begin{aligned} z = {\left\{ \begin{array}{ll} x, & \text {for } L < x \le U \\ l, & \text {for } x \le L \\ u, & \text {for } x > U \end{array}\right. } \end{aligned}$$

The probability density function $\text {PDF}_Z(z)$ of the censored variable *Z* is then given by:

5 $$\begin{aligned} \text {PDF}_Z(z) := {\left\{ \begin{array}{ll} \text {PDF}_X(x), & \text {for } L < z \le U \\ \text {CDF}_X(L), & \text {for } z = l \\ 1-\text {CDF}_X(U), & \text {for } z = u \end{array}\right. } \end{aligned}$$

In the case that *Z* is censored at only one side, its probability function is defined for left censoring as

6 $$\begin{aligned} \text {PDF}_Z(z):= {\left\{ \begin{array}{ll} \text {PDF}_X(x), & \text {for } z > L \\ \text {CDF}_X(L), & \text {for } z = l \\ \end{array}\right. } \end{aligned}$$

and for right censoring as

7 $$\begin{aligned} \text {PDF}_Z(z):= {\left\{ \begin{array}{ll} \text {PDF}_X(x), & \text {for } z \le U \\ 1-\text {CDF}_X(U), & \text {for } z = u \end{array}\right. } \end{aligned}$$

## Modeling truncated data in Stan

As the CDF and the CCDF are needed to model truncated or censored data, we recently extended the diffusion model family in Stan by these functions. Remember that the cumulative distribution function is defined as the probability *P* that *X* takes a value less than or equal to an evaluated value *x*: $\text {CDF}(x) = P(X\le x)$. Furthermore, the *complementary cumulative distribution function* is defined as the complement of the CDF: $\text {CCDF}(x) = 1-\text {CDF}(x)$. CDF and CCDF for reaction time distributions under the diffusion model are, however, traditionally defined slightly differently in terms of so-called *defective* distribution functions as explained in the following.

For this purpose, we discriminate between the terms *(left/right) rt-bound* to refer to the (left/right) response-time bound in the response window, and the terms *response-0-* and *response-1 boundary* for the lower and upper response boundary, respectively, of the diffusion process.

Consider first the basic four-parameter diffusion model. Let *a*, *w*, *v*, and $t_0$ be the diffusion model parameters as introduced above, and let *x* be an observed reaction time. It is important to highlight that, usually, the PDF of a random variable sums up or integrates to 1. This also means that the CDF converges to 1 as *x* increases. In the diffusion model, we see a split for the data belonging to the response-0 boundary and the response-1 boundary. This means that we can define the probability density function and the cumulative distribution function for the response-0 boundary, $\text {PDF}_0$ and $\text {CDF}_0$, and for the response-1 boundary, $\text {PDF}_1$ and $\text {CDF}_1$. One possibility to implement the functions is as *defective* functions. That is, not the individual PDFs and CDFs but the sum $\text {PDF}_0 + \text {PDF}_1$, or $\text {CDF}_0 + \text {CDF}_1$ integrates to 1 or converges to 1, respectively. In this case, the cumulative distribution functions converge to the probability to hit the response-boundary: $\text {CDF}_1(\infty \mid a, w, v)=P(a, w, v)$ for the response-1 boundary and $\text {CDF}_0(\infty \mid a, w, v)=\text {CDF}_1(\infty \mid a, 1-w, -v)=P(a, 1-w, -v)$ for the response-0 boundary, where *P*(*a*, *w*, *v*) is the probability that the diffusion process terminates at the responsee-1-boundary (see Eq. 9). It also follows that the defective complementary cumulative distribution function can be written as $\text {CCDF}_1(x\mid a,w,v) = P(a, w, v)-\text {CDF}_1(x\mid a,w,v)$ for the response-1 boundary and $\text {CCDF}_0(x\mid a,w,v) = P(a, 1-w, -v)-\text {CDF}_0(x\mid a,w,v)$ for the response-0 boundary.

In the following, we introduce the definition of the cumulative distribution function for the response-1 boundary. There are two expressions of the CDF of decision times: one that supports efficient computation of its values for relatively large times, and the other one is more attuned to small times. The formula for the large-time CDF of decision times (excluding the additive reaction time components summarized in $t_0$ for the time being) at the response-1-boundary is stated as follows (adapted from response-0 boundary definition in Hartmann & Klauer, 2021):

8 $$\begin{aligned} \text {CDF}_1(x\mid a, w, v)&:= P(a, w, v) - \exp (va(1-w)\nonumber \\&\quad -\frac{v^2x}{2}) \text {CDF}_l(x\mid a, w, v), \end{aligned}$$

where *P*(*a*, *w*, *v*) is the probability to hit the response-1-boundary, defined as

9 $$\begin{aligned} P(a, w, v) = {\left\{ \begin{array}{ll} \frac{1-\exp (2vaw)}{\exp (-2va(1-w)) - \exp (2vaw)}, & \text {for } v\ne 0 \\ w, & \text {for } v=0, \end{array}\right. } \end{aligned}$$

and

10 $$\begin{aligned} \text {CDF}_l(x\mid a, w, v) = \frac{2\pi }{a^2}\sum _{k=1}^{\infty }\frac{k\sin (k\pi (1-w))}{v^2+(k\pi )^2/a^2}\exp (-\frac{k^2\pi ^2x}{2a^2}). \end{aligned}$$

The formula for the small-time CDF at the response-1-boundary is stated as follows:

11 $$\begin{aligned} \text {CDF}_1(x\mid a, w, v)&:= \exp (va(1-w)\nonumber \\&\quad -\frac{v^2x}{2}) \text {CDF}_s(x\mid a, w, v), \end{aligned}$$

where

12 $$\begin{aligned} \text {CDF}_s(x\mid a, w, v) = \sum _{k=0}^{\infty }(-1)^k\phi \bigl (\frac{a(k+w^*_k)}{\sqrt{x}}\bigr )\times \nonumber \\ \bigl (R\bigl (\frac{a(k+w^*_k)+vx}{\sqrt{x}}\bigr )+R\bigl (\frac{a(k+w^*_k)-vx}{\sqrt{x}}\bigr )\bigr ), \end{aligned}$$

where $w^*_k=(1-w)$ for *k* even, $w^*_k=w$ for *k* odd, and *R* is Mill’s ratio (see section 1 in the supplementary materials of Hartmann & Klauer, 2021; Mitrinović, 1970). The CDF for the response-0-boundary is $\text {CDF}_0(x \mid a, w, v) = \text {CDF}_1(x\mid a, 1-w, -v)$.

From here, it is possible to compute the CDF and CCDF taking into account additive reaction time components $t_0$ as well as the CDF and CCDF for the seven-parameter diffusion model, which also includes the intertrial variabilities for $t_0$, *v* and *w*, where needed. The latter step requires numerical integration in some cases.

For these reasons, the Eqs. (1) to (3) for the density of the truncated data also have to be adapted for the diffusion model. Let *L* denote the left rt-bound and *U* denote the right rt-bound of a response window.

Then, the density of truncated data from response boundary $\text {resp} \in \{0,1\}$ can be formulated as follows:

13 $$\begin{aligned}&\text {PDF}_{\text {resp}}(x \mid L<X\le U, a, w, v) = \\ \nonumber&\frac{\text {PDF}_{\text {resp}}(x \mid a, w, v)\cdot \mathbb {I}_{\{L<x\le U\}}}{\bigl (\text {CDF}_0(U \mid a, w, v)+\text {CDF}_1(U \mid a, w, v)\bigr ) - \bigl (\text {CDF}_0(L\mid a, w, v)+\text {CDF}_1(L\mid a, w, v)\bigr )} \end{aligned}$$

The density of left-truncated data can be formulated as follows:

14 $$\begin{aligned}&\text {PDF}_{\text {resp}}(x \mid L<X, a, w, v) \nonumber \\&= \frac{\text {PDF}_{\text {resp}}(x \mid a, w, v)\cdot \mathbb {I}_{\{L<x\}}}{1-\bigl (\text {CDF}_0(L \mid a, w, v)+\text {CDF}_1(L \mid a, w, v)\bigr )}, \end{aligned}$$

and the density of right-truncated data can be formulated as follows:

15 $$\begin{aligned}&\text {PDF}_{\text {resp}}(x \mid X\le U, a, w, v) \nonumber \\&= \frac{\text {PDF}_{\text {resp}}(x \mid a, w, v)\cdot \mathbb {I}_{\{x\le U\}}}{\text {CDF}_0(U \mid a, w, v)+\text {CDF}_1(U \mid a, w, v)} \end{aligned}$$

Next, we describe how to define a truncated model in the model block of a .stan-file. For a detailed description of the other .stan-file blocks (data-, and parameters-block) see Henrich et al. (2023).

As of Stan version 2.35.0, the seven-parameter version of the diffusion model is available in Stan as described in Henrich et al. (2023).^2^ The three additional parameters in the seven-parameter diffusion model comprise the inter-trial variability in the relative starting point, called $s_w$, in the non-decision time, called $s_{t_0}$, and in the drift rate, called $s_v$ (see Henrich et al., 2023, for more information). For a reaction time *x* at the response-1- boundary, this full model can be called with the following command:
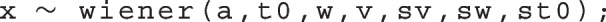


or
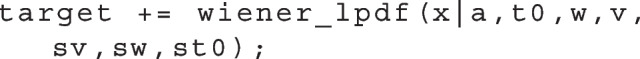


For a reaction time at the response-0-boundary, replace *w* by $1-w$ and *v* by $-v$.

All smaller models can be called by fixing one or more parameters to 0. For example, a model without the inter-trial variability in the relative starting point looks as follows:
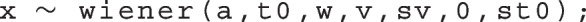


or
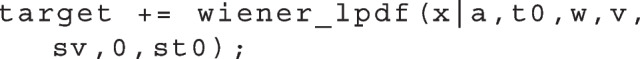


The four-parameter model can be called by setting all inter-trial variabilities to 0:
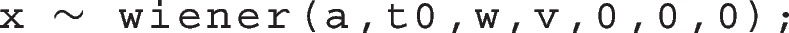


or
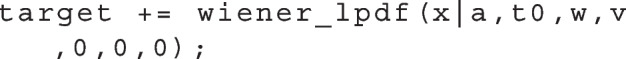


As the functions are implemented defectively, a truncated diffusion model cannot be calculated with the truncation functor *T*[, ] (see Stan Development Team, 2023b). This means the function call: x $\mathtt {\sim }$ wiener(...)T[L,U] does not work the way it is supposed to. When the truncation functor is called in Stan, Stan searches for a CDF implementation internally. In the case of the diffusion model, Stan would find the CDF, but is not aware of its defective implementation and calculates the computations as if it were a non-defective CDF. This causes misleading and incorrect results. Therefore, to implement the truncated model, write out Eq. 13 on the log-scale with left_bound = L and right_bound = U, where wiener_lcdf() calls the logarithmized CDF of the diffusion model at the response-1- boundary:
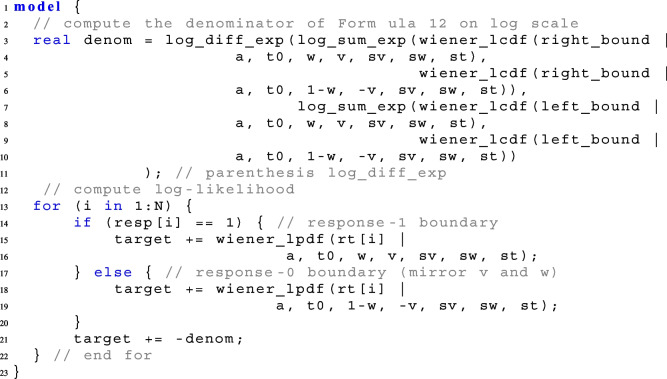


How to call a truncated model within the parallelization routine of reduce_sum or with truncation to only one side (in line with Eqs. (14) and (15)) is described in Appendix A.

## Modeling censored data in Stan

For the censored model, we distinguish two cases: In the first case, the responses of the censored trials are known, but the reaction times are not known. In the second case, neither the responses nor the reaction times of the censored trials are known. Note that the second case differs from a truncated model in the fact that the number of censored trials is still known. Consider first the case where the response is known even for censored data.

To model such data in Stan, the left and right rt-bounds, left_bound and right_bound, respectively, are handed over in the **data block**, as well as a vector censored that tracks whether a trial is censored $(=1)$ or not $(=0)$, and counts of trials censored at the left rt-bound and counts of trials censored at the right rt-bound for each response in $\{0,1\}$. There are four such count variables: N_cens_left_0, N_cens_left_1, N_cens_right_0, N_cens_right_1:
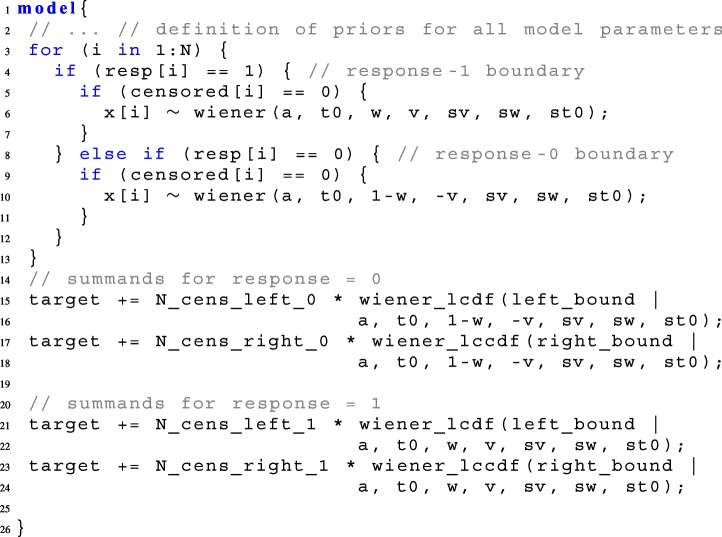


When data are censored at only one side, omit the lines for the other side in the code.

When data consist of many conditions, it is sometimes more convenient to loop over all trials instead of using count variables as described above, using the following notation and code. A vector containing the information whether a trial is censored or not, here censored, needs to be handed over in the **data block**. This vector splits the data into three bins: all trials i with censored[i]=0 are censored below the left rt-bound, all trials i with censored[i]=1 fall between the rt-bounds, and all trials i with censored[i]=2 are censored above the right rt-bound. For non-censored trials, the log-PDF is computed, for left censored trials, the log-CDF is computed, and for right censored trials, the log-CCDF is computed:
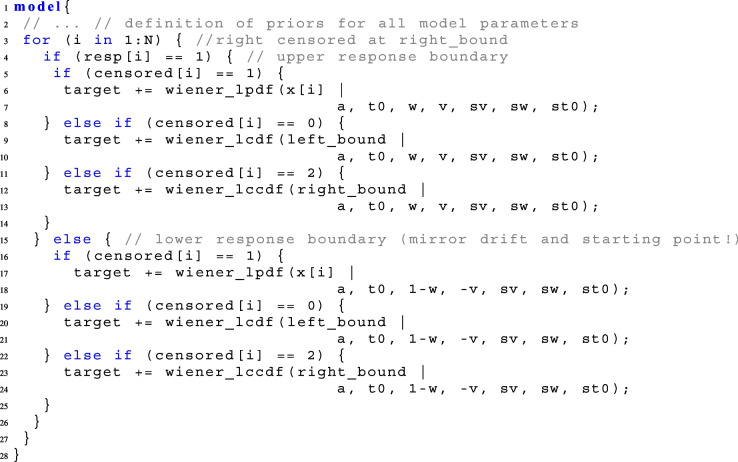


When the data are censored to only one side, omit the case that is not needed. Note that this block can be inserted in the definition of the parallelization function, partial_sum_fullddm(), as defined in Appendix A.^3^

Censoring sometimes includes the response (i.e., it is known that the reaction time in a trial fell outside the response window, but which response was given is unknown). One method that has been used to model such data has involved inferring the numbers of missing responses of either kind from the observed relative frequencies of the two responses (e.g., Pleskac et al., 2018). This approach has the problem that quite specific assumptions on the missing data have to be made (namely, that the proportions of the two kinds of responses are the same for responses within and outside the response window).

We recommend a principled approach that uses the cumulative distribution functions and their complements to provide the likelihood of censored data. As before, let *L* be the left rt-bound, and *U* the right rt-bound, and consider decision times without inter-trial variabilities for the sake of simplicity. It follows that the likelihood $p_l$ of observing a left-censored data point is given by

16 $$\begin{aligned} p_l(a, w, v) \!=\! \text {CDF}_0(\text {L} \mid a, w, v) \!+\! \text {CDF}_1(\text {L} \mid a, w, v), \end{aligned}$$

whereas the likelihood $p_r$ of a right-censored data point is given by

17 $$\begin{aligned} p_r(a, w, v) \!=\! \text {CCDF}_0(U\mid a, w, v)\!+\!\text {CCDF}_1(U\mid a, w, v). \end{aligned}$$

See the following code for an example of Stan code implementing this second case of censoring. This model call deals with the problem of unknown responses by computing the probability of choosing the response-1- or response-0 boundary outside the response window. Here, the CDF and/or the CCDF are required, depending upon whether there is only left-censoring, right-censoring, or censoring both to the left and to the right. The following code shows the **functions block** for a model that is right-censored using the function partial_sum_fullddm() for parallel computations. Combine this block with the **model block** in the example in Appendix A:
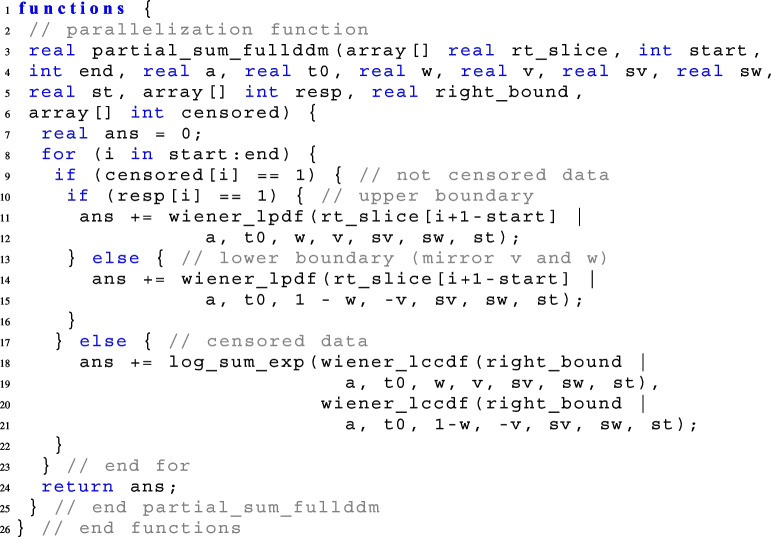


## Validating the new implementation

In this section, we present two consistency checks for the new methods for analyzing truncated and censored data: First, a simulation study to test parameter recovery, and second, a simulation-based calibration study (SBC, Talts et al., 2018) to show the correctness of the implemented algorithm. Both studies have an analogous design as the consistency checks for the implementation of the (non-truncated, non-censored) diffusion model in Stan (see Henrich et al., 2023).

We chose a typical experimental design and priors based on findings in the literature, drew the true parameters from distributions that coincide with these priors, and simulated data using the true parameters. We simulated data that are truncated with a right rt-bound as well as data that are censored with a right rt-bound (in the following referred to as *truncated analysis* and *censored analysis*, respectively). These both correspond to a task with a response deadline in reaction time experiments. We then fitted the data with the appropriate (truncated or censored) model using the parameter distributions underlying data generation in the simulation process as priors. Finally, we analyzed results with respect to recovery and with respect to simulation-based calibration.

### Design

**Fig. 2:**
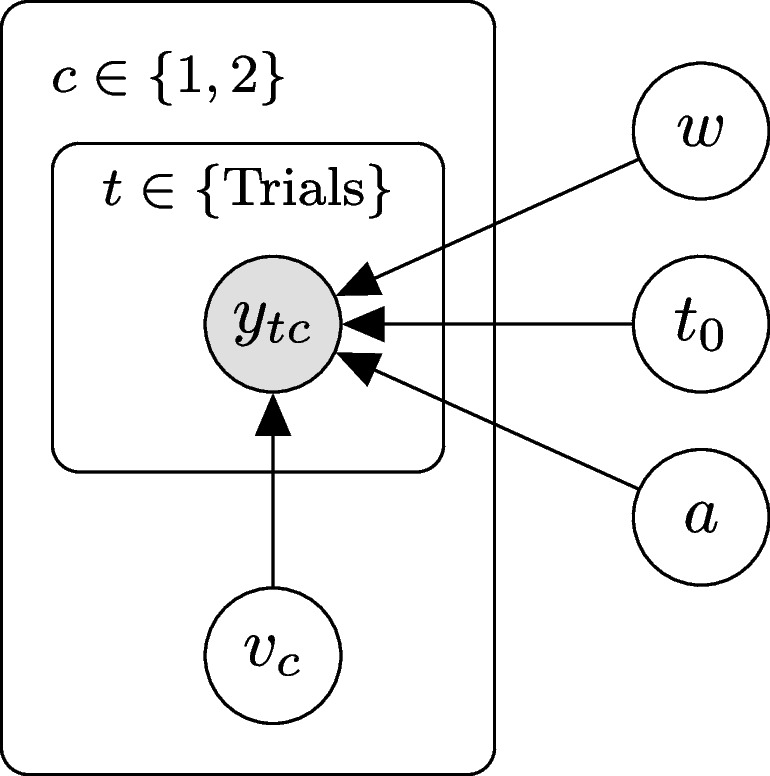
Graphical model representation in the simulation study. *Note.* Each data point $y_{tc}$ (comprising of reaction time and response) within trial *t* and condition *c* depends on the four diffusion parameters. The drift rate varies between conditions. This results in five parameters to estimate

The simulated datasets comprise trials from two conditions, representing two different stimuli, where Condition 1 has positive, and Condition 2 negative *drift rate*. All other parameters are shared across conditions. For reasons of feasible computational time, we simulated data from a non-hierarchical four-parameter model, instead of a seven-parameter model. This is a common design in reaction time experiments (e.g., Arnold et al., 2015; Johnson et al., 2020; Ratcliff & Smith, 2004; Voss et al., 2004). A graphical model representation is given in Fig. 2.

### Ground truths, priors, and parameter distributions underlying data generation

The true parameters for the simulation study, denoted as the *ground truths*, are randomly drawn from parameter distributions which coincide with the priors used in the model and are shown in Table 1.

The choice of the priors and therefore also of the parameter distributions underlying the data generation are based on typical ranges of parameter values as reported in the literature. Specifically, the distributions for *a* and *w* are based on Wiecki et al. (2013, Fig. 1 in the Supplements), the parameter distribution for $t_0$ is based on Matzke and Wagenmakers (2009, Table 3), and the parameter distribution for *v* is the one used in Wiecki et al. (2013)^4^. To simulate the two conditions with different drift rates, we drew two values from the drift rate distribution and multiplied the second value with $-1$, such that in Condition 1, the drift rate is directed to the response-1 boundary and in Condition 2 to the response-0 boundary.

**Table 1:** Parameter distribution for data generation in the simulation study

Parameter	Prior / Data-generating
	parameter distribution
*a*	$\mathcal {N}(1,1) \text { T}[0.5,3]$
*v*	$\mathcal {N}(2,3) \text { T}[0,5]$
*w*	$\mathcal {N}(0.5,0.1) \text { T}[0.3,0.7]$
$t_0$	$\mathcal {N}(0.435,0.12) \text { T}[0.2,1]$

*Note.* $\mathcal {N}$ = normal distribution; $\text {T}[., .]$ = truncation

### Datasets

Following Henrich et al. (2023), we drew 2000 ground truths from the data-generating parameter distributions for the truncated analysis and another 2000 ground truths for the censored analysis. Then, for each analysis, we simulated two datasets for each ground truth: one comprising 100 trials (50 per condition) and one comprising 500 trials (250 per condition). This results in four simulation studies, each comprising 2000 datasets (truncated: 100 and 500 trials, and censored: 100 and 500 trials).

Data were simulated in R (R Core Team, 2021) using the sampling method sampWiener() of the package WienR (Hartmann & Klauer, 2021), which allows one to sample responses and reaction times from truncated diffusion model response time distributions with a right rt-bound. All three inter-trial variabilities were set to 0.^5^ For the truncated analysis, the rt-bound was set to 0.91s. To obtain this value, we first simulated 2000 datasets without a right rt-bound. Next, we determined for each dataset the $80\%$ quantile, that is, an individual right-bound rt-value that splits the specific dataset into $80\%$ less than that value and $20\%$ greater than that value. Finally, we took the mean of all these individual right-bound rt-values to obtain a general right rt-bound for this simulation study, meaning that all datasets in the two truncated studies are truncated above 0.91. This results in 100 and 500 trials, respectively, where each trial has an rt-value less than 0.91s. Note that there is no information on the actual number of truncated trials.

For the censored analysis, the information on the number of trials that are above the rt-bound is included in the model. Here, we first simulated data without any rt-bound. In a second step, we labeled each trial according to whether it had a reaction time below or above the right rt-bound of 0.91, then discarded the reaction time for reaction times above the rt-bound and counted for each of the two drift rate conditions how many of these censored trials had response 0 and 1, respectively.

### Method configuration

Analyses were run on the high-performance computing cluster in Karlsruhe, Germany, BwUniCluster2.0^6^, within the framework program bwHPC. For each analysis, we ran four chains (as recommended by Vehtari et al., 2021). Chains were computed in parallel, and each chain was parallelized on up to 15 cores via the Stan internal parallelization routine reduce_sum(). The method parameter max_treedepth was set to 5 to speed up the sampling process, while still preserving good convergence.

We started computations with 500 warmup and 250 sampling iterations per chain. When results did not converge satisfactorily with this setting, we repeated the analysis of this dataset with increased sampling iterations until all convergence criteria (see below) were met.

### Recovery study

#### Convergence and diagnostics

It is recommended to check some convergence criteria before analyzing the results of the estimation process (e.g., Vehtari et al., 2021). Among these criteria are the *effective sample size*, $N_\text {eff}$, and a convergence measure, $\hat{R}$.

The *effective sample size* is a measure of how many independent samples contain the same amount of information as the dependent samples obtained by the sampling process. It is recommended that the rank-normalized effective sample size is greater than 400, $N_\text {eff}>400$, for each model parameter (Vehtari et al., 2021). The $\hat{R}$ value is a measure of convergence and should be less than 1.01, $\hat{R}<1.01$ (Vehtari et al., 2021).

We checked these two criteria for each dataset and reanalyzed those datasets that did not meet the criteria with more *sampling iterations* until all datasets met the criteria. Thus, all effective sample sizes are above 400 and all $\hat{R}$ values are below 1.01.

#### Recovery

**Fig. 3:**
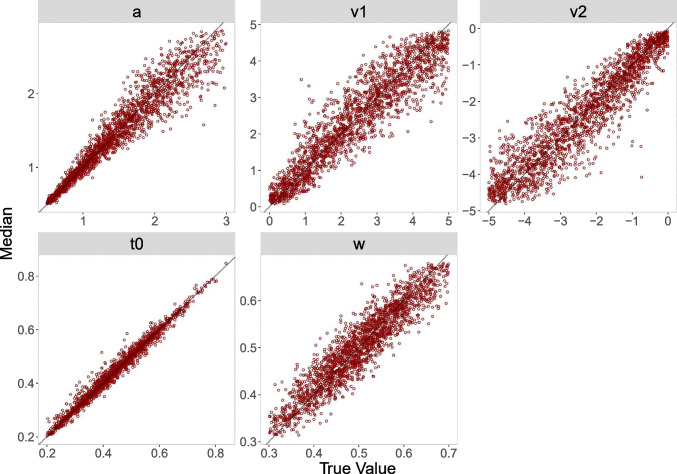
Diagonal plot between posterior median and true value for 100 trials for the truncated analysis

**Fig. 4:**
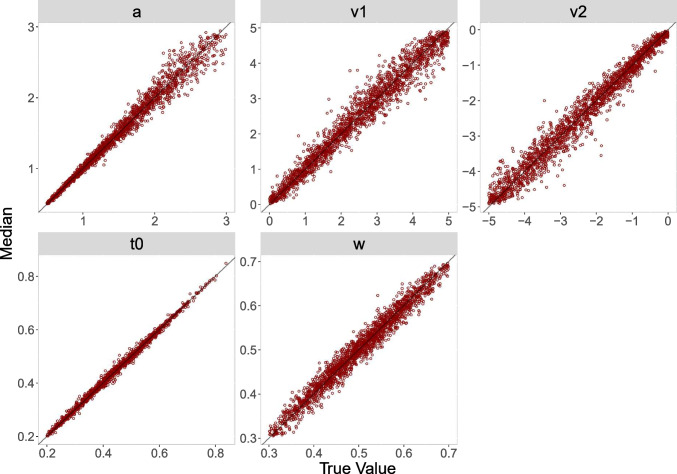
Diagonal plot between posterior median and true value for 500 trials for the truncated analysis

**Fig. 5:**
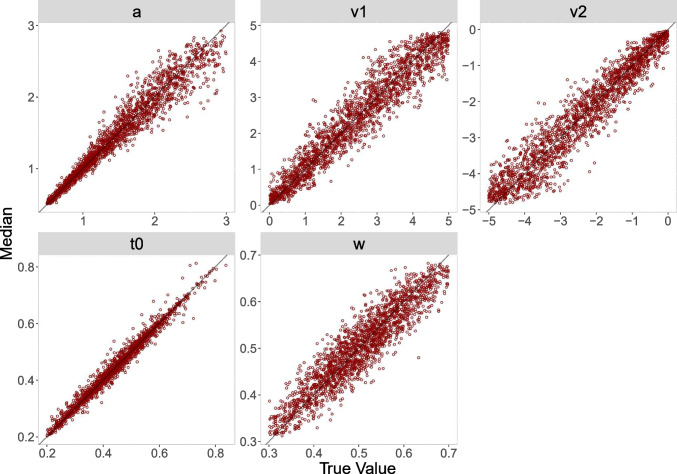
Diagonal plot between posterior median and true value for 100 trials for the censored analysis

**Fig. 6:**
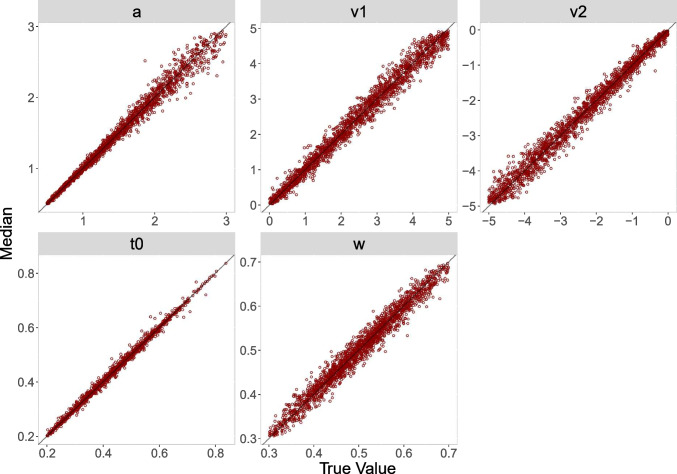
Diagonal plot between posterior median and true value for 500 trials for the censored analysis

To assess recovery, we present three measures: *correlations* between the true values and the posterior median, *coverage*, meaning the percentage of times across the datasets that the true value lies in the $50\%$ and $95\%$ highest density interval (HDI), respectively, and a graphical representation of the *bias* via diagonal plots of the true values against the posterior medians. Results for *correlations* and *coverage* are shown in Table 2 and results for the bias are shown in Figs. 3, 4, 5 and 6.

**Table 2:** Parameter recovery study: Evaluation criteria (correlations, coverage) for parameters estimated from 100 and 500 simulated trials, respectively, for the truncated and the censored analysis

Par.	r	$50\%^{\text {a}}$	$95\%^{\text {a}}$	Par.	r	$50\%^{\text {a}}$	$95\%^{\text {a}}$
— 100 Trials, truncated —				— 100 Trials, censored —			
*a*	.96	50	94	*a*	.97	50	95
$v_1$	.93	50	95	$v_1$	.96	47	95
$v_2$	.93	50	93	$v_2$	.96	47	94
$t_0$	.99	49	95	$t_0$	.99	47	95
*w*	.93	49	95	*w*	.93	48	95
— 500 Trials, truncated —				— 500 Trials, censored —			
*a*	.99	50	95	*a*	.99	49	95
$v_1$	.98	48	95	$v_1$	.99	50	95
$v_2$	.98	49	95	$v_2$	.99	50	95
$t_0$	1.00	49	95	$t_0$	1.00	49	95
*w*	.98	49	94	*w*	.98	52	94

*Note.* Par. = Parameters; r = Correlations (between true parameter values and posterior medians)

$^{\text {a}}$ Percent of simulated datasets with true value in the HDI of this percentage

As can be seen, in both analyses, correlations for all parameters are close to 1 (all greater than .93) and increase in size for datasets with more trials. Moreover, the coverage values closely match the nominal 50% and 95% values for the two HDIs that we monitored.

The diagonal plots for both analyses for 500 trials (Figs. 4 and 6) show smaller biases than the diagonal plots for 100 trials (Figs. 3 and 5). The diagonal plots for the censored analysis (Figs. 5 and 6) are more narrow than the diagonal plots for the truncated analysis (Figs. 3 and 4) for the same trial numbers.

In summary, the results for the recovery study are satisfactory. As expected, the parameter recoveries based on 500 trials are better than those based on 100 trials; that is, correlations are higher, coverage is better, and biases are smaller. Furthermore, recovery results for the censored analysis are slightly better than recovery results for the truncated analysis, suggesting that the information on the upper tails of the diffusion model reaction time distributions present in the censored data is especially helpful in pinning down parameter estimates.

### Simulation-based calibration study

In the Bayesian context, good recovery is neither sufficient nor necessary to demonstrate the validity of a Bayesian algorithm. A more rigorous test is provided by testing simulation-based calibration (SBC, Modrak et al., 2022; Talts et al., 2018). The purpose of an SBC is to show that the implemented algorithm is implemented correctly without errors in the code. This is done by testing whether an algorithm satisfies a consistency condition that it must satisfy if implemented correctly. If this consistency condition is not satisfied, it must be concluded that there are errors in the implementation.

The consistency condition can be stated as follows: If the algorithm is implemented correctly, then the *self-consistency condition* holds:

18 $$\begin{aligned} \pi (\theta ) = \int \int \pi (\theta \mid \tilde{y})\pi (\tilde{y}\mid \tilde{\theta })\pi (\tilde{\theta })\,d\tilde{y}\,d\tilde{\theta }, \end{aligned}$$

where $\tilde{\theta }\sim \pi (\theta )$ are the parameters – referred to as the *ground truth* - sampled from the prior distribution,^7^$\tilde{y}\sim \pi (y\mid \tilde{\theta })$ are the data generated from the model using the ground truth, and $\theta \sim \pi (\theta \mid \tilde{y})$ the posterior samples.

Thus, if sets of parameters $\tilde{\theta }\sim \pi (\theta )$ are repeatedly sampled from the priors, datasets $\tilde{y}$ generated from them, and samples $\theta$ drawn from the posterior distribution given these data, then these samples should follow the same distribution as the samples drawn directly from the prior. This can be tested by computing the *rank statistic* *r* of the prior sample relative to the posterior sample, defined for any one-dimensional function *f* mapping parameters on the real numbers as

19 $$\begin{aligned} r\bigl (f(\theta _1),\dots ,f(\theta _L), f(\tilde{\theta })\bigr ) := \sum _{l=1}^{L}\mathbb {I}\left[ f(\theta _{l})<f(\tilde{\theta })\right] \in [0,L], \end{aligned}$$

where *L* is the number of samples of the posterior distribution, and $\mathbb {I}$ is the indicator function taking the value 1 if the condition in the parentheses holds and the value 0 otherwise. If self-consistency holds, the rank statistic should be uniformly distributed on the set of numbers from 0 to *L*.

The simulation study was designed to test this condition. As the MCMC samples in Stan are autocorrelated, we use a subset of the samples to compute the *rank statistic* for each model parameter and thin the posterior samples according to Algorithm 2 in Talts et al. (2018) to $L=399$ high-quality samples. We set the number of bins in the histogram to 100, such that there are 20 observations expected per bin, across the 2000 simulated datasets. We computed the rank statistic for each model parameter using Equation (19). Following recommendations by Modrak et al. (2022), we also compute the rank statistic for the model’s log-likelihood. The resulting distributions of the rank statistic can be depicted by means of histograms to assess deviations from the uniform distribution. We add a gray band to the histograms that covers $99\%$ of the variation expected for each frequency in a histogram of a uniform distribution, where the $99\%$ expected range of the uniform distribution is determined using the quantile function of the binomial distribution, as the frequency of each bin of the histogram is binomially distributed.

We also calculated the $\chi ^2$-statistics for the differences between expected and observed frequencies of observations per bin for each parameter with expected frequencies given by the expected uniform distribution (i.e., 20 per bin). For each parameter, the observed $\chi ^2$ value is compared to the critical $\chi ^2$ value of 123.23, for $\alpha =.05$ with $df=99$ (number of bins minus 1).

#### Results and discussion

**Fig. 7:**
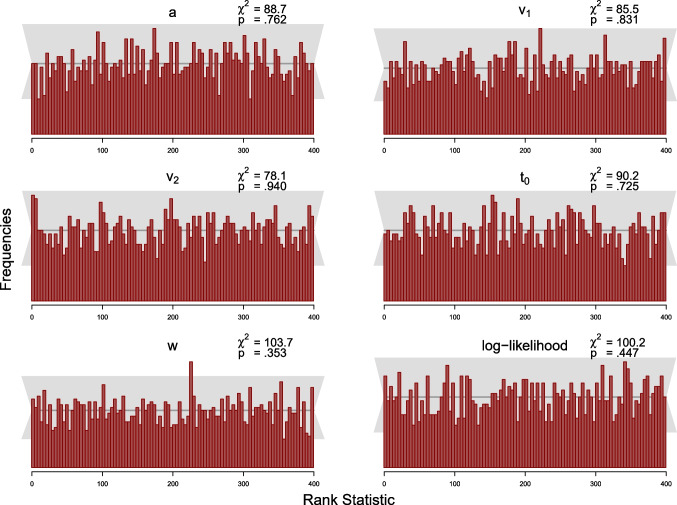
Histograms of the rank statistic for 100 trials for the truncated model. *Note.* The histograms indicate no issues as the empirical rank statistics (*red*) are consistent with the variation expected of a uniform histogram (*gray*)

**Fig. 8:**
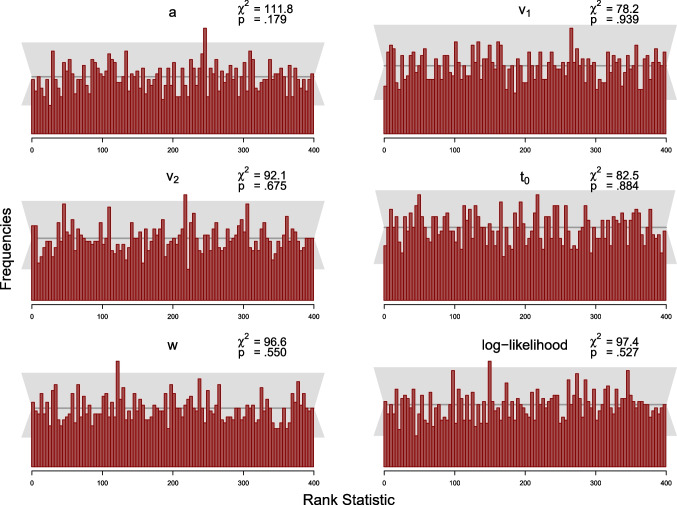
Histograms of the rank statistic for 500 trials for the truncated model. *Note.* The histograms indicate no issues as the empirical rank statistics (*red*) are consistent with the variation expected of a uniform histogram (*gray*)

**Fig. 9:**
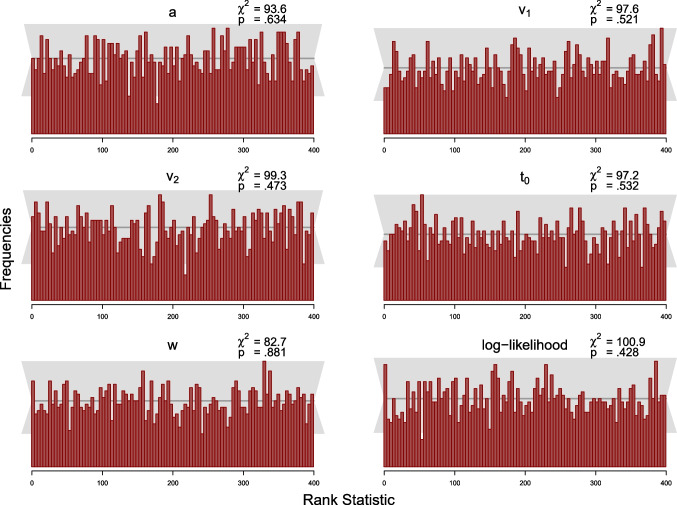
Histograms of the rank statistic for 100 trials for the censored model. *Note.* The histograms indicate no issues as the empirical rank statistics (*red*) are consistent with the variation expected of a uniform histogram (*gray*)

**Fig. 10:**
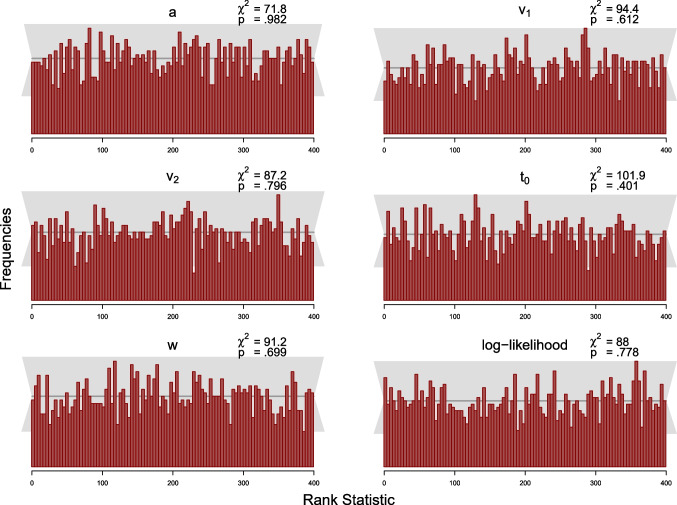
Histograms of the rank statistic for 500 trials for the censored model. *Note.* The histograms indicate no issues as the empirical rank statistics (*red*) are consistent with the variation expected of a uniform histogram (*gray*)

We present results from the SBCs for 100 and 500 trials for the truncated and the censored analyses, respectively, via histograms of the rank statistics (see Figs. 7, 8, 9 and 10). Visual inspection yields that none of the histograms shows systematic variation from the uniform distribution. This means that there is no clear pattern in the histograms that would indicate a bias in the implemented algorithm as described by Modrak et al. (2022) and Talts et al. (2018). Furthermore, all $\chi ^2$-statistics testing for uniformity are non-significant at the $5\%$ level for both analyses.

To sum up, we conclude that there is little indication in these analyses suggesting that the new implementation might be implemented incorrectly.

## Application with First-Person Shooter Task data

As mentioned in the beginning, reaction time experiments with response windows are typical experiments in which truncated and censored data are produced. Ulrich and Miller (1994) advise to include truncation or censoring in the model if data are truncated or censored, respectively. For example, the effects of truncation can alter mean and median reaction times by $10\%$ or more, independent of the exact distribution, and are therefore as large as those of many common experimental manipulations (Ulrich & Miller, 1994)^8^. In the case of diffusion modeling, if right-censoring or truncation is not accounted for, response times appear faster than they truly are, which will in turn impact the parameter estimates; for example, by increasing the absolute magnitude of the estimated drift rates (Pleskac et al., 2018).

To demonstrate functionality of our new implementation, we reanalyze real data from an experiment operationalizing the First-Person Shooter Task (FPST, Correll et al., 2002). We chose to reanalyze data of Study 1 and Study 2 by Pleskac et al. (2018). These datasets suit our purposes due to the following reasons: (a) data and information about the model are freely available online, (b) the design includes a response window that censors data to a right rt-bound, and (c) the authors perform a diffusion model analysis (hierarchical, basic four-parameter model).

### The First-Person Shooter Task

As already mentioned in the Introduction, the FPST is used to study racial bias in shoot/don’t-shoot decisions. Participants see pictures showing a person (Black target or White target) and an object (gun or tool). They are instructed to press the *shoot* key if the target is armed and the *not shoot* key if the target is unarmed. Typical findings are that participants are faster and more accurate to correctly decide "shoot" for Black targets than for White targets and slower and less accurate to correctly decide not to shoot in the case of unarmed Black targets than unarmed White targets (e.g., Amodio et al., 2004; Correll et al., 2002, 2007; Greenwald et al., 2003; Johnson et al., 2017; Payne et al., 2002).

### Study 1 by Pleskac et al. (2018)

Pleskac et al. (2018) investigate the influence of skin color on the decision to shoot using the FPST with different response deadlines and manipulations. In Study 1, targets were shown in a neutral context and a relatively liberal response deadline of 850 ms was used. In only 3% of the trials was the response deadline exceeded so that Study 1 exemplifies a situation in which we would ideally see little effect of whether the model includes censoring or truncation or neither.

**Fig. 11:**
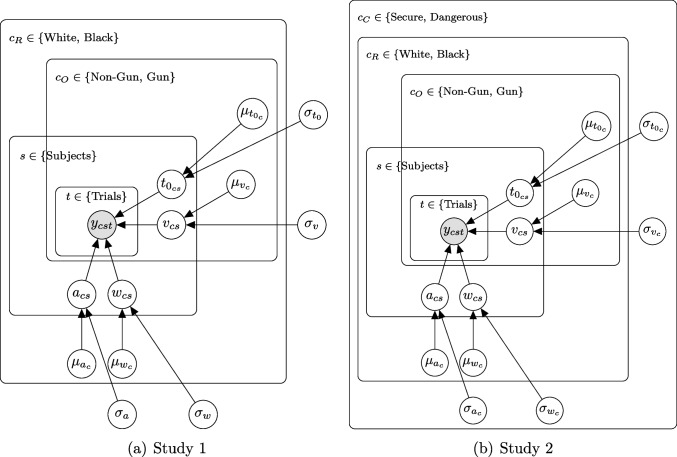
Graphical model representation of the models used in Pleskac et al. (2018). *Note.* The *Context* condition, $c_C$, is a between-subject manipulation, the *Race*, $c_R$, and *Object*, $c_O$, conditions are within-subject manipulations. Subindex *c* refers to the combination of conditions belonging to the plates in which the subindex is located. *y* denotes the data comprising reaction time and response

The authors use a hierarchical censored basic model to analyze data. The *relative starting point*, *w*, and the *boundary separation*, *a*, are allowed to vary across race, but stay constant for the object (gun or tool), so that there are two group-level *w* parameters and two group-level *a* parameters, one per race (Black vs. White). *Drift rate* and *non-decision time* are also allowed to vary as a function of object so that there are four group-level parameters for each of drift rate and non-decision time. A graphical model representation is displayed in Fig. 11(a).

#### Censored data in this study

Data in Study 1 were censored. That is, neither observed response nor response time was recorded for trials in which the response was made outside the response window (i.e., did not occur within 850 ms after stimulus onset). The authors built censoring into the model (using the method described by Kruschke, 2015, Chap. 25.4), see Footnote 1. This approach requires the information to which the response boundary a missing rt-value belongs. To impute the missing response value, the authors used a heuristic way: They imputed missing responses so as to match the observed relative frequency of these responses for gun and non-gun objects for each subject, collapsing across the conditions (Pleskac et al., 2018, Supplementary material).

#### Methods

As Pleskac et al. (2018) provide all data and models online^9^, we first reran the JAGS analysis in the same way the authors did. In a second step, we ran several Stan models. For all models, we choose the same priors as these authors and define four different models: (a) a basic hierarchical model without truncation or censoring, called *basic*, (b) a censored basic hierarchical model, using the responses that were heuristically inferred by the authors, called *censored*, (c) a censored basic hierarchical model, using a principled approach based on the complementary cumulative distribution function (CCDF) to deal with the missing responses as per Eq. (17), called *censored with CCDF*, and (d) a truncated basic hierarchical model, called *truncated*.

The basic model is the baseline model without any accommodation for censoring or truncation. Trials outside the response window are simply omitted from the analyses. The data are thereby analyzed as if there had been no such trials – an analysis that is inconsistent with the implemented response deadline.

For the censored model, we replace missing responses by the responses that were inferred by Pleskac et al. (2018). We expect the results for this model to coincide with the JAGS analysis.

Heuristically inferring the missing responses, as done by Pleskac et al. (2018) relies on strong assumptions about the missing values, namely that correct and error responses would have occurred in the same proportions above the right rt-bound as they did occur inside the response window. As this assumption need not hold in data that stem from a diffusion process, the censored model with CCDF explores an alternative, principled way to deal with missing responses that does not require additional assumptions about the distribution of the missing response values. Instead, we compute the probability of ending at the response-1 boundary or response-0 boundary (and thus, of a correct response or an error response) after the response deadline has passed (Eq. (17)) and multiply the data likelihood by this value for each response outside the response window. This approach correctly encodes the information implied by the event that a response does not occur prior to the deadline in the model’s likelihood function and may be more appropriate when no information on the distribution of the missing response values is available (see above for an implementation of such a model). Because there was little reason to believe that the heuristic assumption required in Pleskac et al.’s (2018) approach would be grossly violated in the present case, we expect this more principled model to perform similarly to the censored model and the JAGS analysis.

Finally, for the truncated model, unlike for the censored model, the number of trials that fall outside the response window remains unknown to the model. Like for the censored model with CCDF, the analysis based on this model is consistent with the implemented response window. It is, however, not informed by the information on the observed number of trials outside the response window and may therefore estimate parameters with somewhat greater uncertainty (expressed in, for example, larger highest density intervals), but should otherwise yield similar parameter estimates as the censored model with CCDF.

#### Results

**Fig. 12:**
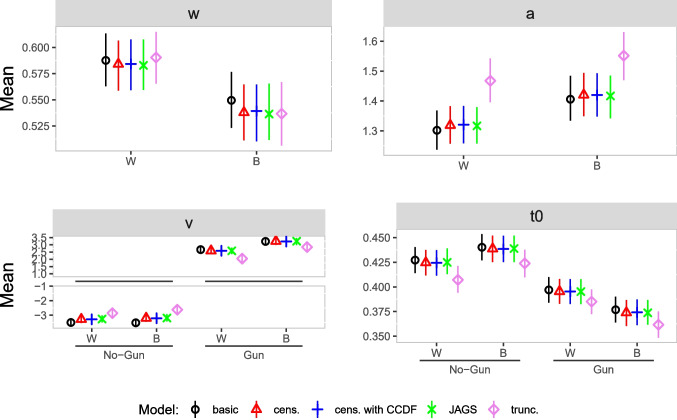
Parameter estimates reanalysis Study 1. *Note.* Posterior means (*dots*) and $95\%$ HDI (*bars*) for the group-level parameter estimates of the diffusion model in each condition for the reanalysis of Study 1; W = White, B = Black, cens. = censored, trunc. = truncated

Figure 12 shows the results of our reanalysis. All four parameters in each condition are displayed. The four Stan models are displayed as a black circle - basic model, red triangle - censored model, blue plus - censored model with CCDF, and violet diamond - truncated model. The results displayed as green cross belong to the JAGS analysis.

We make some observations:

1. The results for the censored model closely match those for the JAGS model. This means that JAGS and Stan behave similarly when applied with the same data and model.
2. The censored model with CCDF deviates little from the censored model and the JAGS model. This suggests that the heuristic assumption built into the censored model (error rates are the same for responses outside the response window as within the response window) is not grossly violated for the present data and model.
3. The basic model shows minor deviations from the censored models in the relative starting point and the boundary separation. As only $3\%$ of the data are censored, it is reasonable to expect similar results from the basic and the censored models.
4. Surprisingly, results for the truncated model deviate more substantially from the other models’ results in nearly all parameters. With this model, the boundary separation is estimated to be larger in both conditions, whereas the drift rate and non-decision time are estimated to be smaller in all conditions. Below, we discuss additional analyses aimed at understanding the cause of this unexpected pattern of results.

### Study 2 by Pleskac et al. (2018)

In Study 2, the target persons are shown in either a neutral or a dangerous context in a between-subjects manipulation of context. The study design thus comprises two within-subject manipulations (race: White/Black, and object: No-Gun/Gun), and one between-subject manipulation (context: neutral/dangerous). Like in Study 1, there was a response deadline, which was set to 630 ms in this study, leading to censoring for 10% of the data.

The authors again use a hierarchical censored basic model to analyze data. In this model, the *relative starting point*, *w*, and the *boundary separation*, *a*, are allowed to vary as a function of race and context, but stay constant for the object. *Drift rate* and *non-decision time* were additionally allowed to vary as a function of object. A graphical model representation is displayed in Fig. 11(b).

#### Methods

As in Study 1, we first reanalyze data using JAGS with the same model and data as provided by the authors. Next, we analyze data with the four Stan models described above.

#### Results

**Fig. 13:**
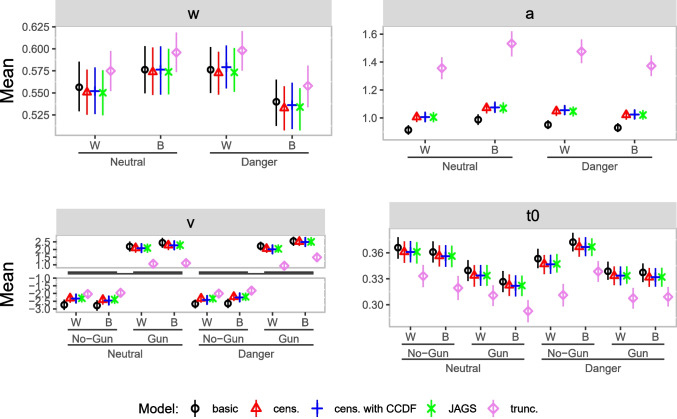
Parameter estimates reanalysis Study 2. *Note.* Posterior means (*dots*) and $95\%$ HDI (*bars*) for the group-level parameter estimates of the diffusion model in each condition; W = White, B = Black, cens. = censored, trunc. = truncated

Figure 13 shows the results of our analysis. We make some observations:

1. The results for the censored model again closely match those for the JAGS model.
2. The censored model with CCDF deviates little from the censored model and the JAGS model. This again suggests that the heuristic assumption built into the censored model (error rates are the same for responses outside the response window as within the response window) is not grossly violated for the present data and model.
3. The basic model shows some deviations from the censored models in all parameters. The estimates for boundary separation are generally smaller than estimates in the censored models, and absolute values of drift rates, one relative starting point and some non-decision times are estimated a little bit larger than in the censored models. That deviations from the basic model are somewhat more pronounced than in Study 1 was to be expected given the higher rate of censoring (10% vs. 3%).
4. Parameter estimates based on the truncated model again show unexpectedly substantial deviations from the results obtained with the other models in all parameters. With this model, the relative starting point and boundary separation are estimated to be larger in all conditions, whereas the drift rate and non-decision time are estimated to be smaller in all conditions. Next, we turn to analyses shedding some light on this unexpected pattern of results.

### What is the cause of the discrepancies between the truncated and the censored models?

We did not expect substantial discrepancies between the truncated and the censored model with CCDF. Both models take the use of a response window into account, the major difference being that the censored model makes use of the information of how many trials had no response within the response window, whereas the truncated model ignores this information. We therefore expected estimates from the truncated model to be associated with some more uncertainty than those of the censored model, but little systematic deviation between the analyses based on the two models. This is in fact the pattern of results exemplified by the simulation study.

Clearly, this expectation was not borne out, as evidenced by the substantial deviations between the analyses based on the truncated and the censored model, which in turn matched the analyses based on the basic model relatively closely in comparison. This implies that the assumptions from which our expectation was derived are wrong – our major assumption was that a diffusion process generated the analyzed data. But how exactly does this lead to the observed discrepancies?

We reasoned that the data must violate the diffusion model assumptions in such a way that the violations capitalize on the difference between the basic and the censored model, on the one hand, and the truncated model, on the other hand, to bring out the observed pattern of discrepancies in the modeling results.

Analyses by the basic and the censored model are constrained by the proportion of censored trials (i.e., trials for which responses did not occur in the response window). For the censored model, the observed proportion of such trials is directly encoded in the data likelihood; for the basic model, such trials are nonexistent, and the data likelihood encodes the (wrong) information that such trials did not occur; their proportion is implicitly assumed to be zero.

In contrast, for the truncated model, the proportion of trials without response in the response window is an unknown. The model acknowledges the existence of a response window, but remains agnostic about the percentage of trials without responses within the response window. These trials do not inform the data likelihood of the truncated model in any way (other than by allowing for their existence).

In fitting the data, the truncated model is therefore constrained only by the reaction time distribution and responses observed within the response window. The basic and censored model must also attempt to fit the proportion of censored trials (implicitly set to zero in the basic model and equaling the observed proportion of such trials in the censored model).

Thus, discrepancies between the basic and censored model, on the one hand, and the truncated model, on the other hand, might reflect that the proportion of censored trials may be incompatible with the distribution of reaction times and responses within the window. Therefore, the truncated distribution might not be well described by diffusion model parameters, which describe the proportion of censored trials well and vice versa. Specifically, if the distributions of reaction times and responses within the window, when considered in isolation as done by the analysis via the truncated model, are best fit by diffusion model parameters which overall predict larger proportions of censored trials than the observed proportion and the zero proportion implied by the basic model, we expect the outcome of the analysis by the truncated model to differ substantially from the outcome of analyses which also try to fit these proportions.

If this analysis of our results pattern holds true, we should see (a) higher rates of predicted proportions of censored trials for the truncated model analysis than for the other model analyses, along with (b) a better account of the distribution of reaction times within the window by the truncated model than by the other models.

#### Observed and predicted frequencies of trials without response

Tables 3 and 4 show observed and predicted frequencies of trials without response. For Study 1, all models overestimate the number of these trials for the No-Gun conditions and underestimate their number for the Gun conditions. For Study 2, this pattern is similar for the basic and the censored models. The truncated model overestimates the number of such trials in all conditions. Furthermore, in both studies, the truncated model predicts many more such trials in all conditions than the other models.

#### Observed and predicted RT distributions within the response window

**Fig. 14:**
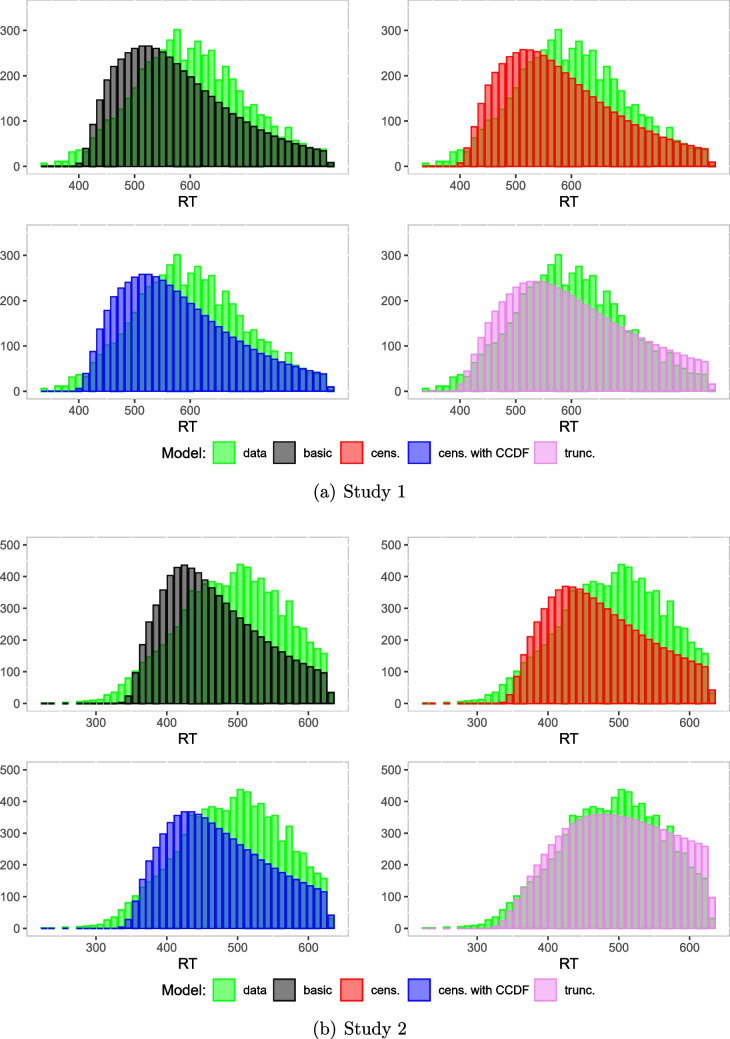
Histograms of predicted reaction times vs. observed reaction times. *Note.* The *green histograms* are based on the data observed by Pleskac et al. (2018) in Study 1 (**a**), and Study 2 (**b**); cens. = censored; trunc. = truncated

Next, we computed predicted reaction times by simulating data from the respective diffusion models using the estimated parameters and again the function sampWiener(). Specifically, we first simulated a large number of datasets based on the samples from the posterior distribution and counted the bin frequencies for each dataset. Then we computed the mean frequency for each bin. The resulting histograms with the mean bin frequencies of the generated datasets and the histogram of the observed data are shown in Fig. 14 for both studies. We observe that the histograms for the basic and the censored models are slightly shifted to the left compared to the observed data. Furthermore, the histogram belonging to the truncated model matches the data histogram best in both studies.

**Table 3:** Observed and predicted numbers of trials without response in Study 1

Model	Conditions			
	W/NG	B/NG	W/G	B/G
basic	172	212	5	1
cens.	221	287	6	1
cens. CCDF	221	286	6	1
trunc.	421	555	19	3
data	52	72	29	15

*Note.* W = White; B = Black; G = Gun; NG = No-Gun; cens. = censored; trunc. = truncated

**Table 4:** Observed and predicted numbers of trials without response in Study 2

Model	Conditions							
	Neutral				Dangerous			
	W/NG	B/NG	W/G	B/G	W/NG	B/NG	W/G	B/G
basic	275	352	30	20	304	315	29	19
cens.	440	517	38	26	468	483	40	21
cens. CCDF	438	515	39	26	463	483	42	22
trunc.	931	1177	178	150	1054	1000	206	96
data	179	170	66	63	163	176	92	62

*Note.* W = White; B = Black; G = Gun; NG = No-Gun; cens. = censored; trunc. = truncated

**Fig. 15:**
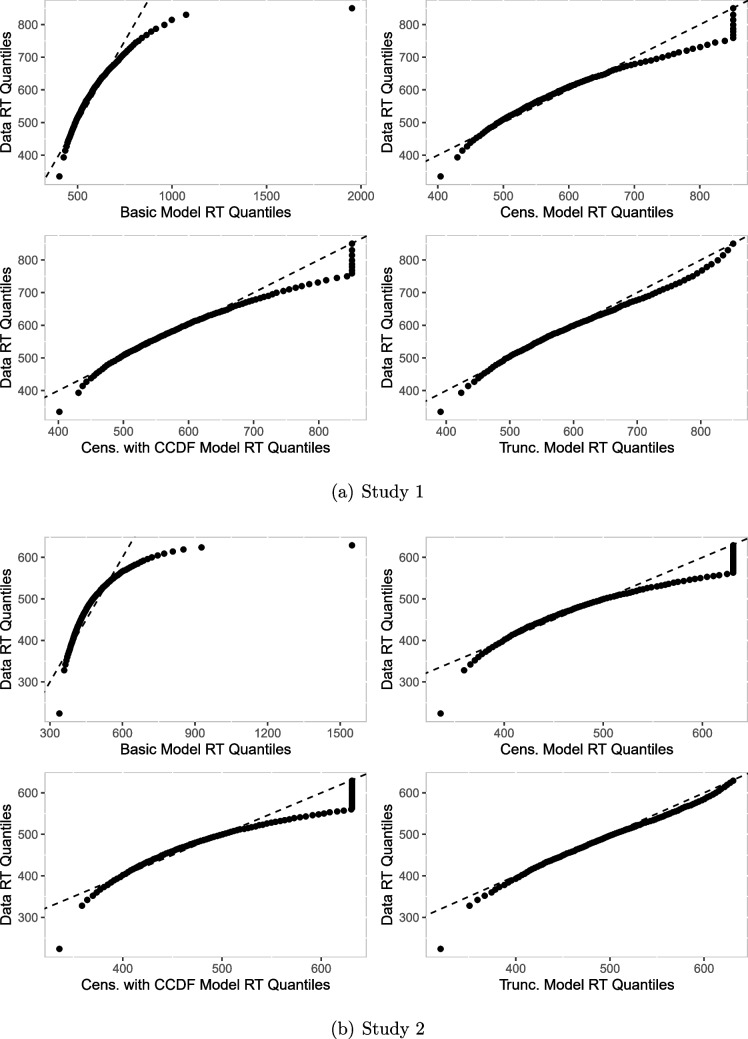
Quantile-quantile plots with predicted vs. observed data quantiles

Similar observations can be made in the quantile-quantile plots in Fig. 15. Points align closely with the diagonal line across all quantiles for the truncated model, showing that explicitly modeling truncation improves fit. For the censored models, points also align closely with the diagonal line in the middle quantiles and deviate slightly from the diagonal line at higher quantiles. For the basic model, there is a substantial deviation from the diagonal line at higher quantiles.

To sum up these analyses, we found support for the above analysis of the causes of the discrepancies between the analyses by the truncated model, on the one hand, and the basic and censored model, on the other hand. It seems to be the case that describing the data from trials with response in the response window by the diffusion model requires parameter values that strongly overestimate the overall proportion of censored trials. The truncated model is not informed by the proportion of censored trials and thereby acquires the flexibility to account for the reaction time distribution within the response window better than the other models at the expense of predicting overall much higher proportions of censored trials than the other models. The other models are constrained by the proportion of censored trials (implicitly set to zero in the basic model and given by the observed proportion in the censored models) and predict much lower proportions of such trials at the expense of less convincing fits of reaction time distributions within the response window.

#### Model selection index: WAIC

As a model-selection index, we compute the Widely Applicable Information Criterion, also known as the Watanabe-Akaike Information Criterion (WAIC, Gelman et al., 2014; Watanabe, 2010). The WAIC is an extension of the Akaike Information Criterion (AIC) and is more appropriate for Bayesian analyses than the AIC. The WAIC estimates the effective number of parameters to adjust for overfitting. Models with smaller WAIC values are to be preferred. Table 5 shows the WAIC values for all models and both studies.

Note that WAIC values can only be meaningfully compared for models fitted to the same data. For the reanalyzed studies, the basic and the truncated model analyze the same datasets without the censored trials, and the two censored models analyze the same datasets that differs from the one used for the basic and truncated model in that it includes information on the censored trials. In comparing WAIC values for the basic and truncated model, the truncated model has a smaller WAIC in both studies, and in comparing WAIC values for the two censored models, the censored model based on the CCDF has the smaller WAIC in both studies.

These observations support our previous findings that the truncated model describes the analyzed data better than the basic model. Furthermore, the censored model based on the CCDF performs better on the data than the censored model based on the heuristic approach, which supports our claim from the beginning for the need for a more sophisticated modeling approach for censored data.

### Discussion

To illustrate the behavior of the new functionality in Stan – modeling truncated and censored data with the diffusion model – we reanalyzed data of Study 1 and Study 2 by Pleskac et al. (2018). For this purpose, we analyzed data with the JAGS model specified by Pleskac et al. (2018) and with four Stan models.

In the first study, only 3% of the trials had no response within the response window. In consequence, we did not expect and did not observe pronounced differences between the analysis by the basic diffusion model without accommodation for the use of a response window and the censored models. In the second study, a stricter response deadline led to 10% of trials without response in the response window. Here, differences between the basic and the censored models were somewhat larger. For example, parameter estimates for boundary separation were smaller without censoring in the model, the non-decision time was larger, and the drift rate was larger in absolute size.

We expected similar patterns of parameter estimates for the analysis by the truncated model as for the censored model. To our surprise, the largest differences were found between the truncated model, on the one hand, and all other models, on the other hand.

We believe we have provided a plausible account of these discrepancies and have corroborated our account with additional analyses. Ultimately, the discrepancy between the analyses using truncated and censored models, along with our additional analyses, shows that the present data are quite pronouncedly incompatible with the assumption that an underlying Wiener diffusion process generates them. Note also that depending upon the pattern of violations of this assumption, we might in fact have seen other patterns of unexpected and surprising results. The takeaway recommendation here might be to routinely implement and report diagnostic model checks in addition to reporting the fitted parameter values to safeguard against surprises stemming from model misfit.

As suggested by an anonymous reviewer, violation of the diffusion model assumptions might stem from a proportion of trials in which responses and reaction times reflect a guessing process or are the result of mind wandering. Alternatively, the response-window procedure itself, which provides feedback if participants’ responses fall outside the window, may have led participants to adapt the data-generating process online, which could also explain the violation of the diffusion model assumptions. Similar remarks pertain to the possibility of outliers in the data. These possibilities need further investigation.

**Table 5:** Goodness-of-fit measure: WAIC

Model	Study 1	Study 2
basic	-8138	-12794
trunc.	-9145	-16157
cens.	-7252	-8517
cens. CCDF	-7310	-9308

*Note.* trunc. = truncated; cens. = censored. Note that values can be compared only between the basic and the truncated model and between the two censored models (see text)

## General discussion

The purpose of this work was to add the functionality to model censored and truncated data in diffusion model analyses in Stan. This involves implementing the cumulative distribution function of reaction time distributions arising from the diffusion model and its complement.

As mentioned in the introduction, truncated or censored data arise in paradigms that use temporal response windows outside of which responding is not possible or for which response times and/or the response falling outside the window are not recorded, as well as a consequence of post-hoc outlier analyses. For censored data, a count of these trials is kept; for truncated data, not even a count is available.^10^ As Ulrich and Miller (1994) elaborated, it is important to build the model used to analyze such data so that it accounts for censoring or truncation if data are censored or truncated. Otherwise, important characteristics of the response-time distributions such as mean, median, standard deviation, or skewness will be estimated incorrectly, biasing model-based analyses. In the case of the diffusion model, the drift rate, for example, will be overestimated when the model does not account for censoring or truncation by an upper response deadline (Pleskac et al., 2018). In order to account for truncation and censoring, we extended the diffusion model implementation in Stan, wiener(), tested the implementation with two consistency checks (recovery and simulation-based calibration) and reanalyzed existing datasets.

We conducted a simulation study assessing recovery from truncated and censored datasets. The results of the recovery studies are satisfactory in terms of correlations, coverage, and bias. Results for the simulation-based calibration studies do not show systematic errors, providing a more stringent test of the correctness of the current implementation than is possible via recovery studies.

We illustrated the new method by reanalyzing data from Studies 1 and 2 in Pleskac et al. (2018). Both of these studies employed response deadlines beyond which no response or response time was recorded. The reanalysis demonstrated that it can make a major difference whether data are analyzed without provision for the response deadline, using a model for censored data, or a model for truncated data (ignoring the information on the number of trials without response before the deadline). As expected, differences between a naive analysis without provision for the response deadline and the censored analysis were small in Study 1, in which most responses occurred prior to the response deadline, and somewhat larger in Study 2 in which the deadline was stricter and more trials occurred without response before the response deadline. These differences were, however, still small in comparison to the discrepancies in parameter estimates obtained from fitting the model for truncated data. Additional analyses suggest that this somewhat surprising result ultimately reflects deviations of the data from the diffusion model.

Our studies are limited by the fact that they were performed for a basic diffusion model with four parameters instead of for the full diffusion model with seven parameters. This limitation reflects the considerable increase in computing time required for fitting the seven-parameter model (Henrich et al., 2023) in a hierarchical design. This renders a large simulation study based on the seven-parameter model unrealistic in terms of required computing times. Future analyses could test the diffusion model on data that are generated from a mixture of different models reflecting guessing or mind wandering to see how robust Bayesian diffusion modeling is against such outliers (see Ratcliff & Tuerlinckx, 2002).

In conclusion, the new features of wiener() produce reliable and competitive results for the basic model and enrich the landscape of diffusion modeling approaches. Using previously published datasets, we demonstrated the functionality of the new implementation and provided hands-on instructions on how to implement a censored or truncated model. We hope that these tools will prove useful for researchers wishing to analyze truncated or censored data with diffusion models.

## Figures and Tables

**Fig. 16:**
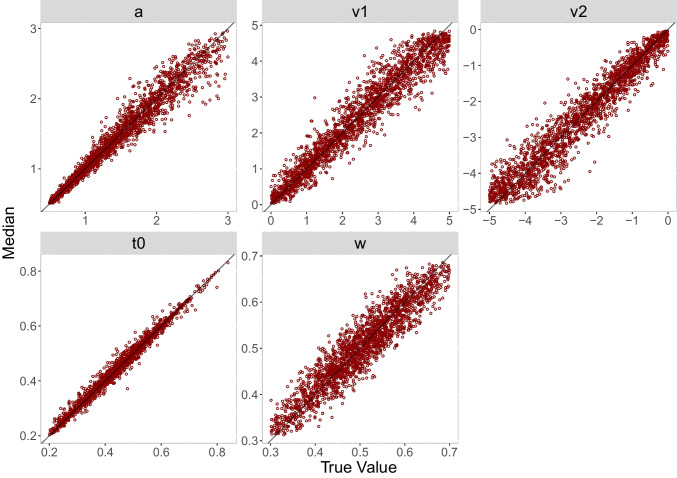
Diagonal plot of posterior median against true value for 100 trials for the four-parameter non-censored analysis

**Fig. 17:**
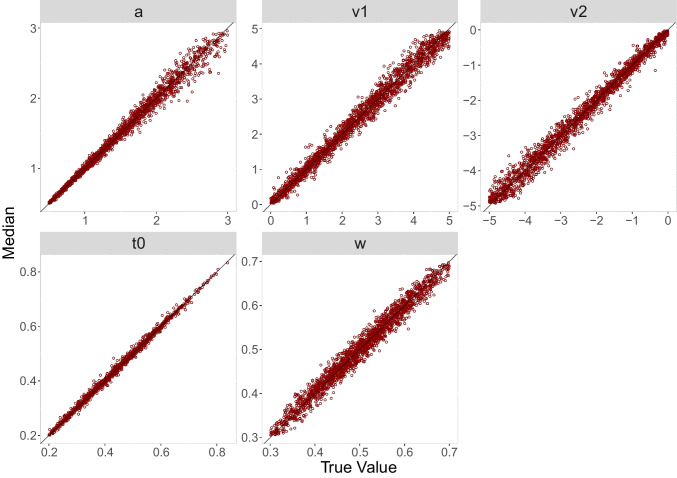
Diagonal plot of posterior median against true value for 500 trials for the four-parameter non-censored analysis

**Fig. 18:**
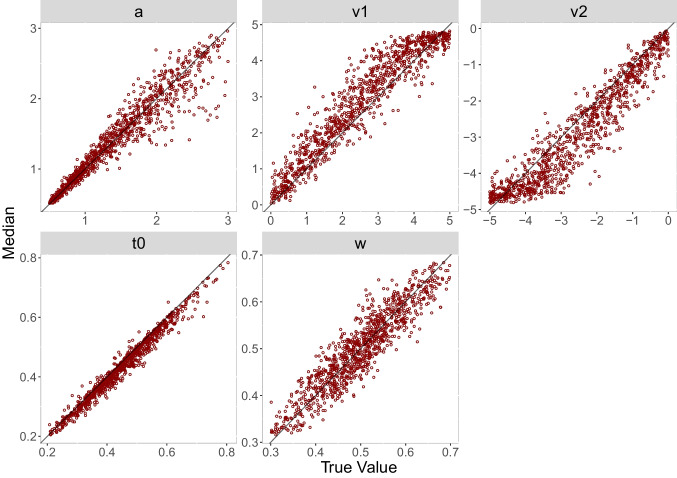
Diagonal plot of posterior median against true value for 100 trials for the seven-parameter non-censored analysis

**Fig. 19:**
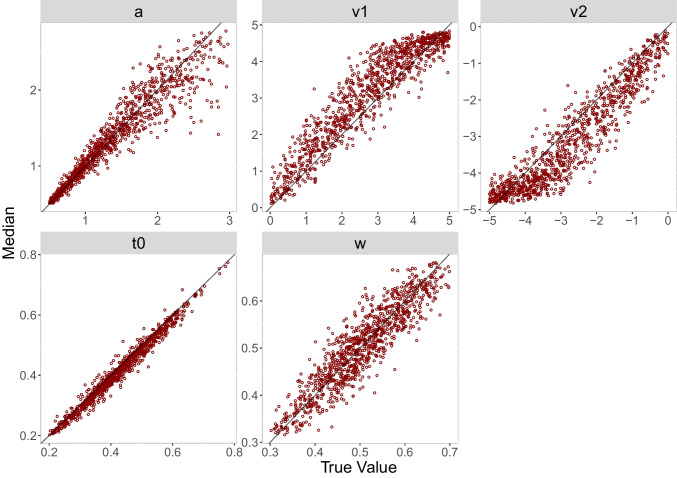
Diagonal plot of posterior median against true value for 100 trials for the seven-parameter censored analysis

**Fig. 20:**
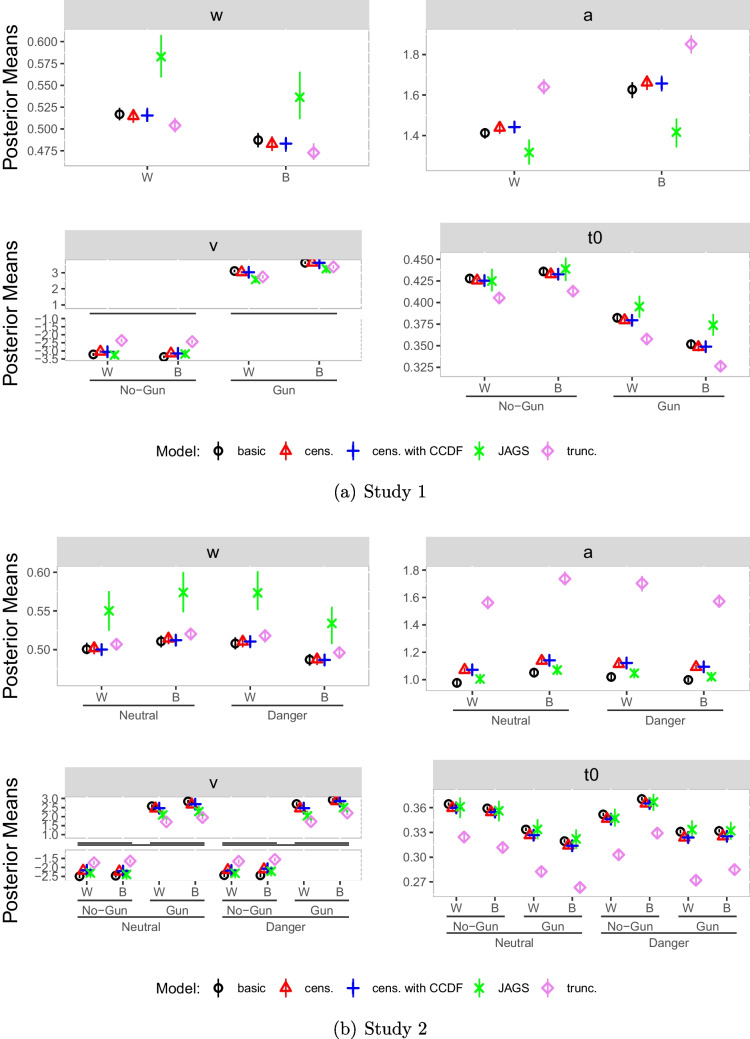
Parameter estimates non-hierarchical reanalysis, one model per person. *Note.* Means (*dots*) and 95% HDI (*bars*) for the group-level parameter estimates of the diffusion model in each condition; W = White, B = Black, cens. = censored, trunc. = truncated

**Fig. 21:**
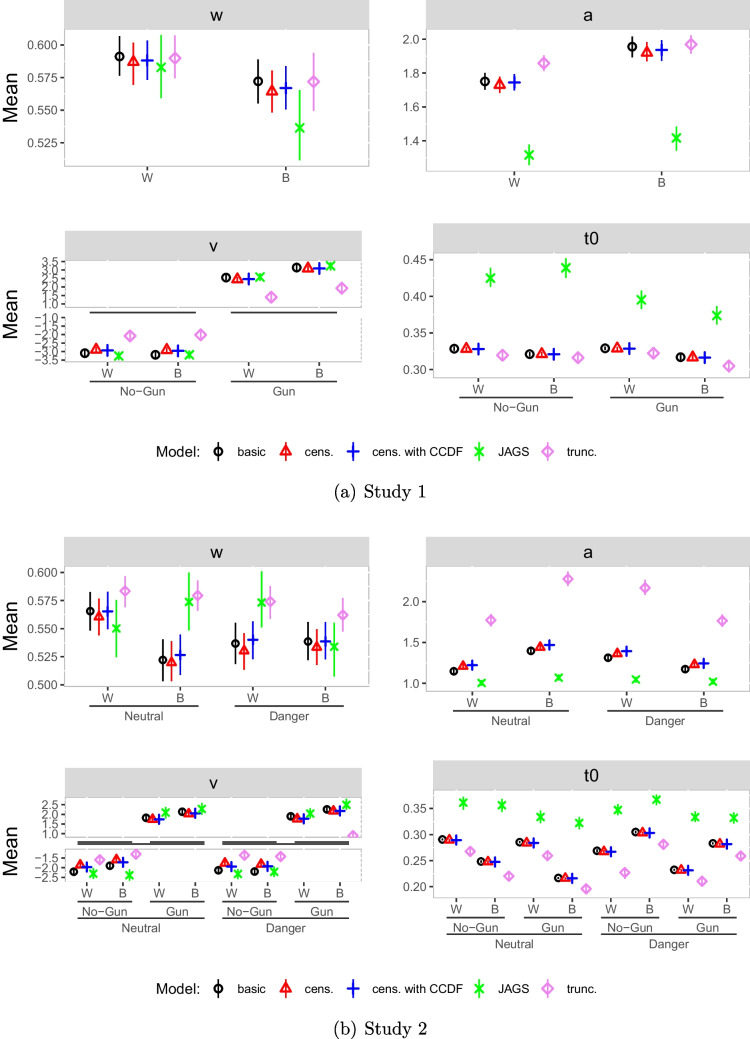
Parameter estimates non-hierarchical reanalysis, one model for all data. *Note.* Means (*dots*) and 95% HDI (*bars*) for the group-level parameter estimates of the diffusion model in each condition; W = White, B = Black, cens. = censored, trunc. = truncated

## Data Availability

All R scripts for the simulations and the empirical analyses, as well as experimental datasets, are available at the Open Science Framework: https://osf.io/vg7zf/.

## Appendix A: Function call truncated model

To use the functionality of parallelizing the estimation process over several cores, the target+= notation has to be used in the **model block** of the Stan file. The two variables left_bound and right_bound are handed over in the **data block** or set directly in the .stan-file. Furthermore, the function that parallelizes the model calls, partial_sum_fullddm(), has to be defined in a **functions block**. Note that in the input data for a Stan analysis, no NA values are allowed. Make sure to delete all trials with missing reaction time values.

The following code corresponds to Eq. (13) on the log-scale:

For data that are only left-truncated, change the selected lines in the above code as follows. This corresponds to Eq.  (14) on the log-scale:

For data that are only right-truncated, change the selected lines in the above code as follows. This corresponds to Eq. (15) on the log-scale:

For a general introduction to Stan, see Stan Development Team (2023a).

## Appendix B: Additional reanalysis of the simulation study data

Following a suggestion by a reviewer, we additionally analyzed the censored datasets from the simulation study with the seven-parameter model with and without censoring and with the four-parameter model without censoring to see whether the seven-parameter model would capture censored data and underlying parameters better.

**Table 6:** Parameter recovery study: Evaluation criteria (correlations, coverage) for parameters estimated from 100 and 500 simulated trials, respectively, for the four-parameter model without censoring, and the seven-parameter model with and without censoring

Par.	r	$50\%^{\text {a}}$	$95\%^{\text {a}}$	Par.	r	$50\%^{\text {a}}$	$95\%^{\text {a}}$
— 100 Trials, 4-p. nc —				— 100 Trials, 7-p. nc —			
*a*	.98	49	95	*a*	.95	53	95
$v_1$	.96	51	95	$v_1$	.95	46	91
$v_2$	.96	48	94	$v_2$	.94	43	90
$t_0$	.99	48	94	$t_0$	.99	41	95
*w*	.93	49	95	*w*	.92	48	96
— 500 Trials, 4-p. nc —				— 100 Trials, 7-p. c —			
*a*	.99	49	95	*a*	.95	52	96
$v_1$	.99	49	95	$v_1$	.94	44	91
$v_2$	.99	51	95	$v_2$	.94	39	89
$t_0$	1.00	49	94	$t_0$	.99	41	95
*w*	.99	50	95	*w*	.91	49	95

*Note.* Par.=Parameters; r=Correlations (between true parameter values and posterior medians), 4-p. nc = four-parameter model non-censored, 7-p. nc = seven-parameter model non-censored, 7-p. c = seven-parameter model censored

$^{\text {a}}$ Percent of simulated datasets with true value in the HDI of this percentage

Due to the high computational demand imposed by fitting the seven-parameter models, we only reanalyzed 1000 datasets in the 100-trial condition for these models each. Therefore, the plots are thinned compared to the four-parameter model plots, which are based on 2000 datasets.

The results for correlations and coverage in 50% and 95% HDI are shown in Table 6. For the seven-parameter model, the inter-trial variabilities are omitted as the comparison focuses on the parameters shared by the four-parameter and the seven-parameter model. The results for assessing the bias in parameter recovery are shown in Figs. 16, 17, 18 and 19.

Results show that the seven-parameter model does not capture the data and underlying parameters better than the four-parameter model in terms of coverage, correlations, and bias. If anything, there appears to be somewhat more bias in the recovery of drift rates under the seven-parameter model, whether censored or not. From these findings, we can conclude that the seven-parameter model with or without censoring does not necessarily fit censored data better than the four-parameter model with censoring included.

## Appendix C: Reanalysis of the Pleskac data with non-hierarchical models

Following another suggestion by a reviewer, we additionally analyzed the datasets of Pleskac et al. (2018) with non-hierarchical models. We examined two different variants of non-hierarchical modeling. This means, in contrast to the analyses shown in the main body, we did not fit the data per condition in a hierarchical manner, but, for the first variant, we fitted each participant separately. We generated the same number of samples from the posterior distribution for each participant. For each parameter, we then averaged the individual parameter estimates from the $i-$th sample across participants for each $i = 1\ldots$ to obtain a sample of the posterior distribution of parameter means under the non-hierarchical model applied jointly and independently to each participant. For the second variant, we fitted all data with a non-hierarchical model as if all data were from one person.

The plots in Fig. 20 show the posterior means and their HDIs for the first variant to which we added the JAGS results from the above hierarchical analyses for the sake of comparison. It can be seen that the non-hierarchical results capture major trends that the JAGS results show. However, for both studies, results show sizeable deviations from the JAGS results in the relative starting point, the boundary separation and the non-decision time. Furthermore, it is also noticeable that the HDIs of the non-hierarchical posterior means are smaller than the HDIs of the population-level parameters of the hierarchical analyses (compare with Figs. 12 and 13), reflecting the fact that hierarchical modeling achieves *partial* pooling, leading to more appropriate assessments of uncertainty in estimation as quantified by HDIs than does either no pooling or complete pooling. Again, the results of the truncated model deviate from the results of the other models as seen before in the hierarchical analyses.

The plots in Fig. 21 show posterior means and HDIs for the second variant, where all data were fit with one model. We also added the JAGS results from the above hierarchical analyses to the plots. In this variant, not all results capture the main trends in the data as the hierarchical analyses. For example, in Study 2, results for the relative starting point deviate for both the neutral and the danger condition, between White and Black targets. Again, in both studies for two of four parameters (boundary separation and non-decision time), results show sizeable deviations from the JAGS results.

To sum up, the results of the non-hierarchical analyses capture main trends in the data for the non-hierarchical models fitted to each participant separately. Therefore, in this variant, one would probably not come to different conclusions regarding the effects of the factors that Pleskac et al. (2018) manipulated in this case. However, when all data are fit with one model as if they were from one person, the non-hierarchical analyses do not capture all trends that the hierarchical analyses capture, and even show opposite trends for some conditions and parameters. This probably reflects the fact that analyzing data with nonlinear models without taking the heterogeneity of the involved data sources into account is known to introduce systematic biases in parameter estimates (e.g., Rouder & Lu, 2005).

## Footnotes

1 In Bayesian analyses, it is possible to model censored data indirectly via a data augmentation step in which the missing reaction times are parameters of an extended model with values imputed in each step of a Markov-chain Monte Carlo algorithm (see Kruschke, 2015, Chap. 25.4). Note, however, that this approach requires further modification in diffusion modeling when not only the reaction time is missing, but also the response itself.

2 Note, however, that its name changed from wiener_full() to wiener() in the course of its recent release in Stan.

3 Also note that no NA values can be handed over to Stan. Hence, fill the missing values with some information, e.g., $\texttt {censored[i]}=42$, for coding NA in the i-th field. Every value is fine, except NA, since the vector censored splits cases, and the censored cases are independent of the concrete reaction time values.

4 Wiecki et al. (2013) based their choice of prior distributions for the diffusion model parameters on values reported in the literature and collected by Matzke and Wagenmakers (2009).

5 A simulation study of the present size would hardly be feasible with the seven-parameter diffusion model, as the required computational time would be very large. As the critical new parts of the code occur in the four-parameter core model, we performed our analyses on the four-parameter diffusion model.

6 https://wiki.bwhpc.de/e/Registration/bwUniCluster

7 For simulation-based calibration, it is important that the parameter distribution underlying the data generation is the same as the prior distribution in the Bayesian model.

8 Ulrich and Miller (1994) examined distributions like the lognormal, Erlangian, and Ex-Gaussian distribution.

9 osf.io/9qku5/

10 Truncation is less frequent in psychological reaction-time experiments than censoring. Consider, as an example, a consumer-psychology study in which the stimuli are different prices presented for an item on sale on the Internet. Customers can decide whether they want to buy one item or two items at a discount. The response times to be modeled are the times to the first purchase. If prices change with a fixed interstimulus interval, we will only be able to register purchases that occur within the interval. If no purchase is registered within the interval, we do not know whether none occurred or whether a purchase occurred, but came too late. In this case, the data on purchase decisions are effectively truncated by the duration of the interstimulus interval.

## Code availability

The code will be available in Stan and is expected to be included in Stan with the next release. Until then, the implementation is available here: https://github.com/Franzi2114/math/tree/feature/issue-2966-Add-7-parameter-DDM-CDF-and-CCDF, and an instruction on how to get started with the new function can be found here: https://github.com/Franzi2114/math_HOW-TO-USE.
